# A Dietary Management System Using Radio-Frequency Identification Technology to Collect Information on Chinese Food Consumption: Development and Feasibility Study

**DOI:** 10.2196/mhealth.7674

**Published:** 2018-08-14

**Authors:** Xiaowei Xu, Ju Wang, Li Hou, Zhen Guo, Jiao Li

**Affiliations:** ^1^ Institute of Medical Information & Library Chinese Academy of Medical Sciences Beijing China; ^2^ School of Biomedical Informatics The University of Texas Health Science Center at Houston Houston, TX United States

**Keywords:** diet records, RFID technology, radio frequency identification device, food consumption, Chinese foods

## Abstract

**Background:**

Dietary management is important for personal health. However, it is challenging to record quantified food information in an efficient, accurate, and sustainable manner, particularly for the consumption of Chinese food.

**Objective:**

The objective of this study was to develop a dietary management system to record information on consumption of Chinese food, which can help in assessing individuals’ dietary intake and maintaining healthy eating behaviors. We proposed to use plates embedded with radio-frequency identification chips to carry Chinese foods and collect food consumption data.

**Methods:**

We obtained food composition and nutrient (eg, carbohydrate, fat, fiber) data from the Chinese Recipe Database and China Food Composition Database. To test the feasibility of the dietary management system at a population level, we applied it to collect data on 489 Chinese foods that were consumed at lunchtime across 7 weeks by 10,528 individuals. To test individual-level output, we selected an individual participant with completed 20-day dietary data for analysis. We examined the system’s nutrient calculation performance by comparing the nutrient values of 3 selected Chinese dishes calculated by our method with the results of chemical measurements.

**Results:**

We collected the dietary intake for a group of 10,528 individuals aged from 20 to 40 years having lunch in a restaurant across 7 weeks. A total of 489 Chinese dishes were identified. We analyzed a specified customer’s diet recordings and broke his or her 20 lunch diet recordings down to ingredients and then to nutrient intake. We compared the nutrient value of a given Chinese dish (eg, garlic puree cooked pork leg) calculated by our method with the results of chemical measurements. The mean absolute percentage deviation showed that our method enabled collection of dietary intake for Chinese foods.

**Conclusions:**

This preliminary study demonstrated the feasibility of radio-frequency identification–based dietary management for Chinese food consumption. In future, we will investigate factors such as preparation method, weight of food consumed, and auxiliary ingredients to improve dietary assessment accuracy.

## Introduction

### Dietary Assessment

Diet is an important risk factor or prevention-related factor for health management and disease treatment. Healthy eating behaviors can help decrease the burden of chronic diseases such as overweight, cardiovascular disease, liver disease, and diabetes [1-4]. Accurate methods to record food consumption and assess dietary intake are essential in health-related research and health care interventions.

Commonly used dietary assessment methods include dietary records, 24-hour dietary recall, food frequency questionnaires, and brief dietary assessment instruments [5]. In the dietary record approach, individuals must record each of the consumed foods in detail (eg, food name, preparation methods) and their amounts measured by scales or estimated by models. To complete a dietary record, individuals are required to describe these details at the time of food consumption. In the 24-hour dietary recall, individuals are required to remember the foods consumed in the past 24 hours. This is traditionally conducted by a trained interviewer in person or by telephone. In the food frequency questionnaire method, individuals are required to report the frequency of their consumed foods from given checklists. Brief dietary assessments are developed for situations that do not require either assessment of the total diet or quantitative accuracy in dietary estimates. For example, brief instruments have been used for population surveillance in the Behavioral Risk Factor Surveillance System [6].

Technical advances in information and communication technology have improved the automatic collection of self-reported dietary data by means such as standardized question sequencing, fasting data processes, and increased flexibility [7]. In recent years, new technology-based dietary assessments have included online (Web-based) methods, mobile methods, and sensor technology, thus assisting clinical studies and nutritional epidemiology research. These new information and communication technology-based efforts are at various stages of development [8]. For instance, the Graphical Food Frequency System was developed to address the food frequency questionnaire’s limitations inherent in paper questionnaires [9]. A mobile app, Diet-A, was developed for self-monitoring dietary intake [10].

### Objective

In this study, we proposed to apply a sensor technology, radio-frequency identification (RFID), to collect information on consumption of Chinese food.

RFID technology enables information interaction or exchange without human intervention and awareness [11]. RFID-based systems have been used in health care environments [12] and the food industry [13,14]. Recently, RFID chips have been embedded in plates for dietary recording [15,16]. Wireless interaction between the RFID-embedded plates and RFID readers facilitates automatic collection of food consumption information with supporting dietary assessment.

In Chinese diets, foods are cooked according to numerous recipes, and are classified as staple foods (eg, rice, steamed buns), cooked dishes (eg, cooked tomato with eggs), and soups (eg, egg drop soup). In this study, the RFID plates were used to carry the Chinese foods. We developed our dietary management system to automatically collect food consumption information (ie, food names and food frequency) but no personal information of the consumers (eg, sex, age, and smoking habits). The system further provided functions of food frequency statistics, food composition overview, and proxy indicators of dietary intake.

## Methods

### Participants

A total of 10,528 participants took part in this study. Participants’ ages ranged from 20 to 40 years, and all had access to a restaurant equipped with a dietary management system for lunch. Each of them was assigned a unique identification (ID) the first time they had meals in the restaurant.

### Dietary Management System Design

**Figure 1:**
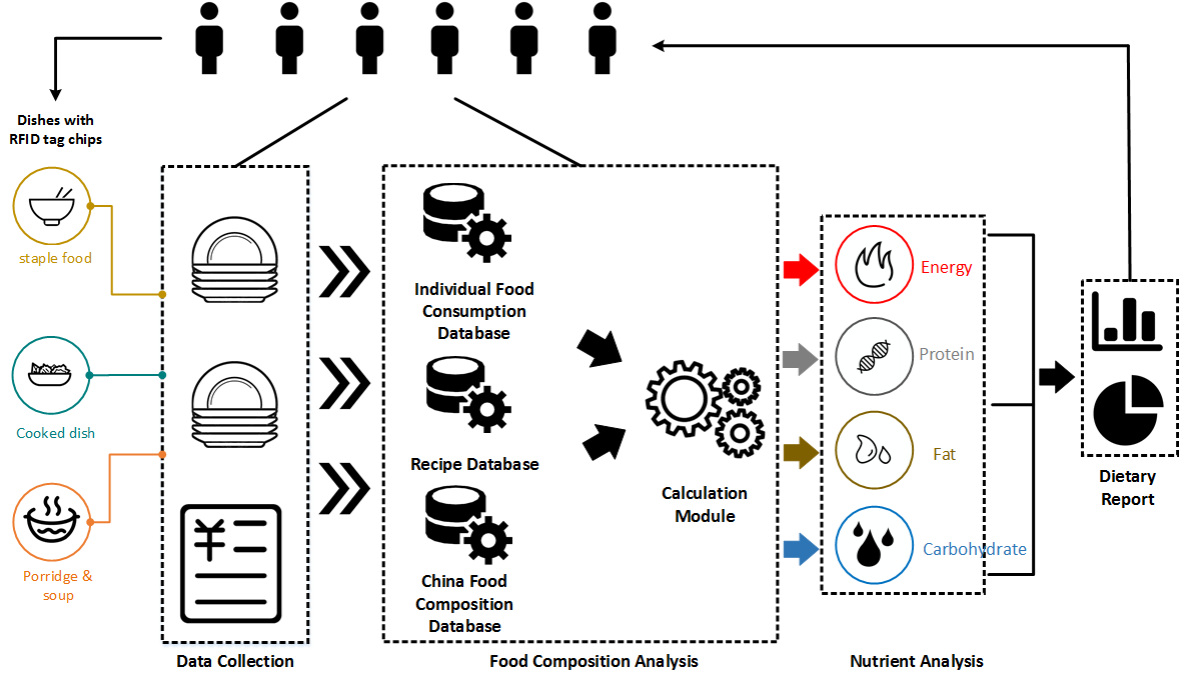
A radio-frequency identification (RFID)–based dietary management system for Chinese foods.

We designed and implemented a dietary management system for the collection of Chinese food information (see Figure 1). It comprises 3 key components: the RFID-based dietary data collection module, the Chinese food composition analysis module, and the nutrient analysis module.

#### Radio-Frequency Identification–Based Dietary Data Collection

We used iPlate (Hangzhou Sovell Technology Development Company Limited), a research and development solution for dietary management, which provides an open platform supporting Chinese nutrient research [17]. A standard iPlate set comprises 3 components: 3 types of plates embedded with RFID chips, which provided information such as what kinds of foods were on the plate and their corresponding weights; a production bench, which can erase and rewrite the RFID chips embedded in the plates, and a settlement bench, with an RFID reading function.

We classified foods into 3 categories, put onto 3 types of plates: staple foods (eg, rice) are on plate 1, cooked dishes (eg, cooked tomato with eggs) are on plate 2, and soups (eg, egg drop soup) are on plate 3. The RFID chips embedded in the plates recorded information about the foods, including their name, price, and weight. When a diner checked out after choosing a plate of food, the RFID reader interacted with the plate, recording food consumption information by connecting the consumer’s ID with the food information.

Each plate contained 1 type of food or dish. The weight of food in each plate was set by the restaurant according to the food itself and the type of plate. Dietary information (cooked food price, food name, consumer ID, and time of meal: breakfast, lunch, or supper) was stored in the individual food consumption database.

#### Chinese Food Composition Analysis

To analyze the composition of the chosen foods, we developed 2 databases: a recipe database and a nutrient intake database.

The recipe database stored the Chinese food recipes, including the food names, main ingredients and their corresponding weights, and preparation methods. The function of the recipe database was to break down the Chinese foods on the RFID plates into their ingredients.

The function of the food composition database was to break down the ingredients into their nutrient components. We used the China Food Composition Database [18,19] as the data resource, which includes 23 nutrients for 1400 food ingredients.

#### Nutrient Analysis

We selected 3 specific Chinese dishes for nutrient calculations. We first broke down their ingredients according to the recipe database. Then, we calculated the dishes’ total nutrients by summing the nutrients of each food ingredient according to the China Food Composition Database. We coded Chinese food in the food composition table. Our previous studies on data representation for food nutritional composition [20] and development of a nutrient content retrieval-oriented search engine for Chinese recipes [21] laid the foundation for the nutrient analysis.

**Figure 2:**
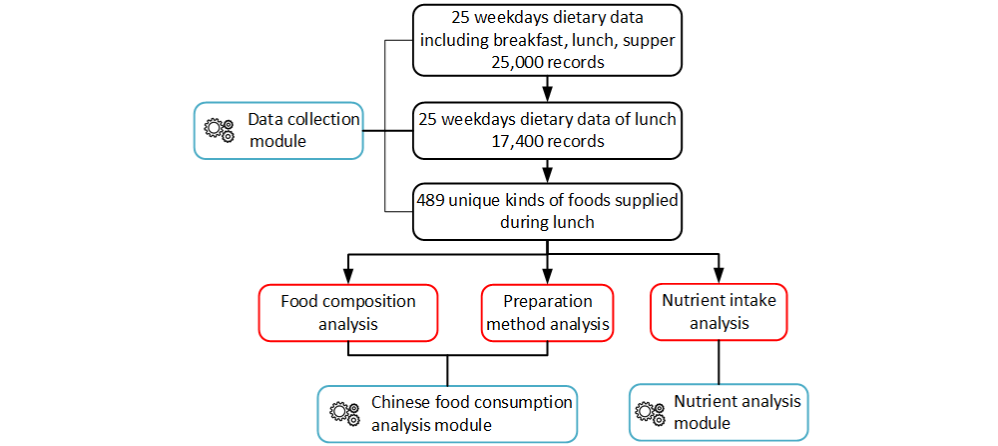
Data selection process for dietary records.

### Data Selection Criteria

We used the following criteria to select RFID-collected data for further analysis, as Figure 2 shows: (1) dietary data records collected at lunchtime, (2) Chinese foods on the RFID-embedded plates, (3) the chosen food recipes included in the recipe database, (4) the chosen food ingredients included in the composition database.

### Feasibility Testing

To test the feasibility of the dietary management system, we applied it to collect data on 489 kinds of Chinese foods, which were consumed at lunchtime by our study participants from January 5 to February 15, 2016. We used these examples to test our system output at the population level, including consumed food distribution and preparation method distribution. To test our system at an individual level, we selected one participant who completed 20-day dietary data.

## Results

### Food Consumption Distribution

**Figure 3:**
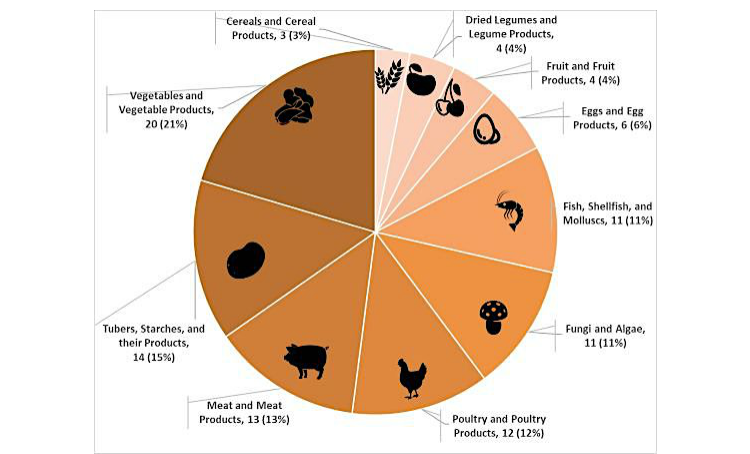
Example of system output: distribution of consumed foods.

We obtained 489 kinds of unique Chinese dishes eaten by diners at lunchtime. The system monitored the distribution of the ingredients of all the dishes provided, as Figure 3 illustrates. We can see that vegetables and vegetable products (eg, tomato, cabbage) made up 21% (20 kinds of vegetables out of 91 kinds of ingredients) of the dishes monitored. Tubers, starches and their products, meat and meat products, poultry and poultry products, fungi and algae, and fish, shellfish and molluscs all account for over 10% of all of the dishes consumed.

### Preparation Method Distribution

Following Chinese dietary habits, we divided the 489 dishes of consumed Chinese foods into 3 types: staple food (83/489, 17.0%), cooked food (391/489, 80.0%), and porridge and soup (15/489, 3.0%).

**Figure 4:**
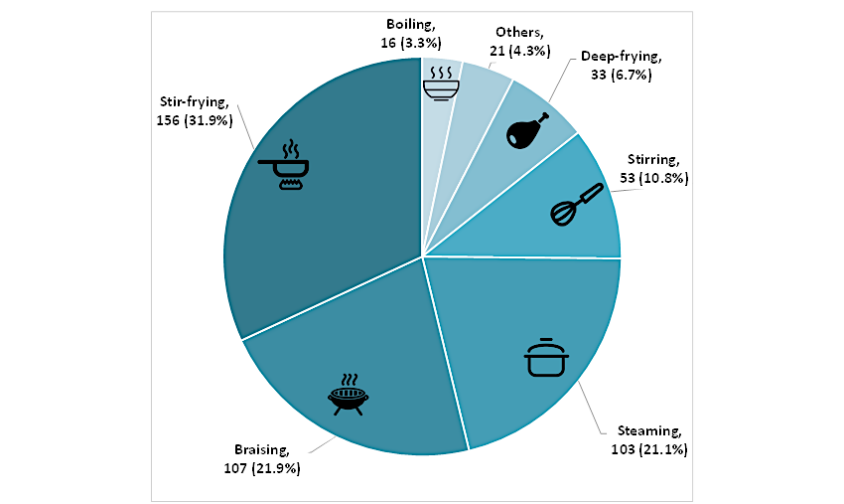
Example of system output: distribution of preparation methods.

The proportion of cooked food is 80% (n=391), which means it is an indispensable component of Chinese food. Based on the cooked food recorded, we analyzed the distribution of preparation methods at the population level (see Figure 4). This group of diners preferred the stir-fried dishes.

### Personalized Nutrient Analysis

**Figure 5:**
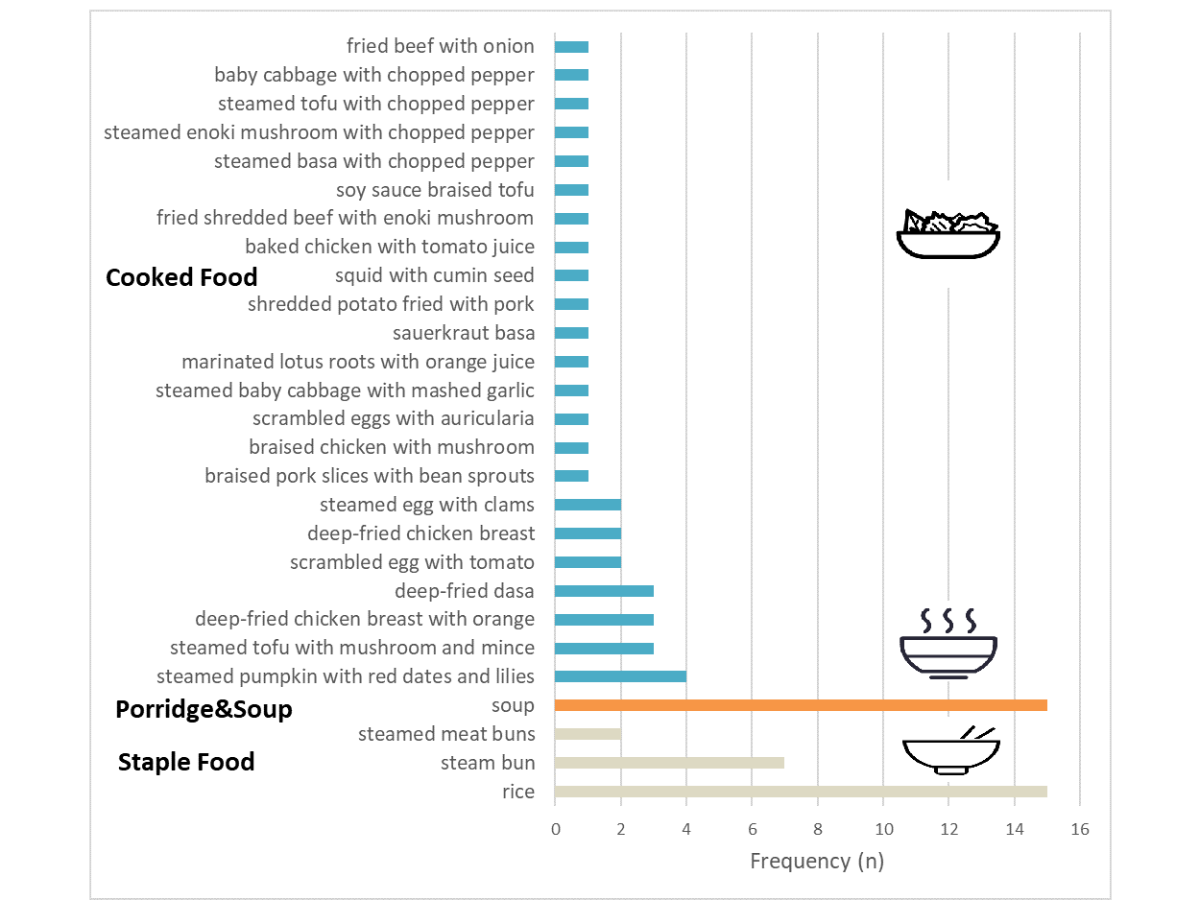
Example of system output: frequency of foods consumed by 1 sample participant.

To demonstrate the individual-level system output, we selected an individual with completed 20-day dietary data for analysis. Figure 5 shows the individual’s consumed food frequency at lunch over 20 days. This person liked to eat rice together with soups, having chosen rice and soup 15 times within the 20 days.

**Figure 6:**
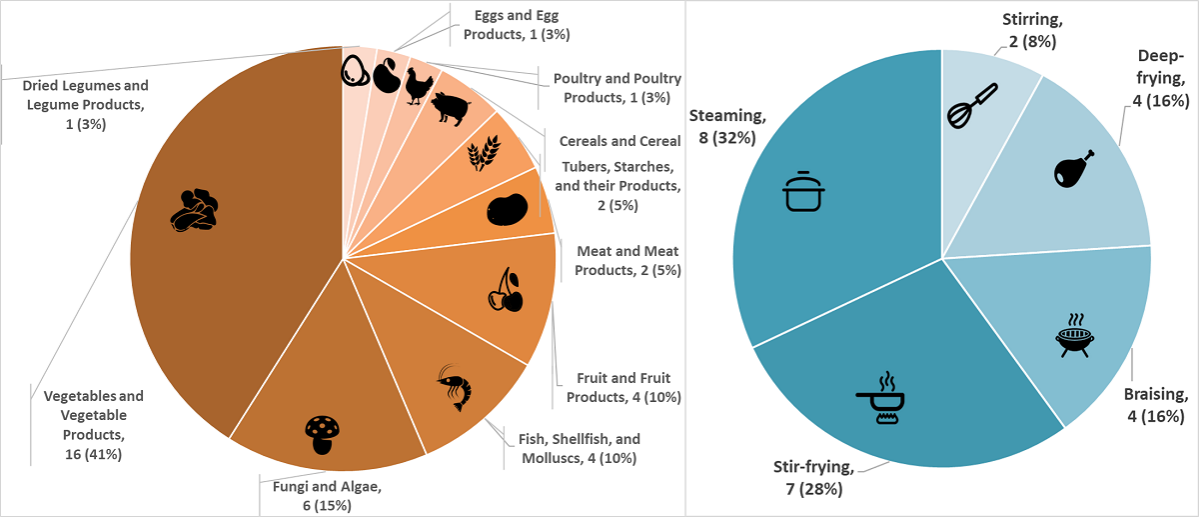
Example of system output: a sample individual’s preferred food composition (left) and preparation methods (right).

Figure 6 shows this sample individual’s eating preferences for food composition (left) and preparation methods (right). Compared with the population-level distribution (see Figure 3 and Figure 4), this individual preferred vegetables and steamed foods.

**Figure 7:**
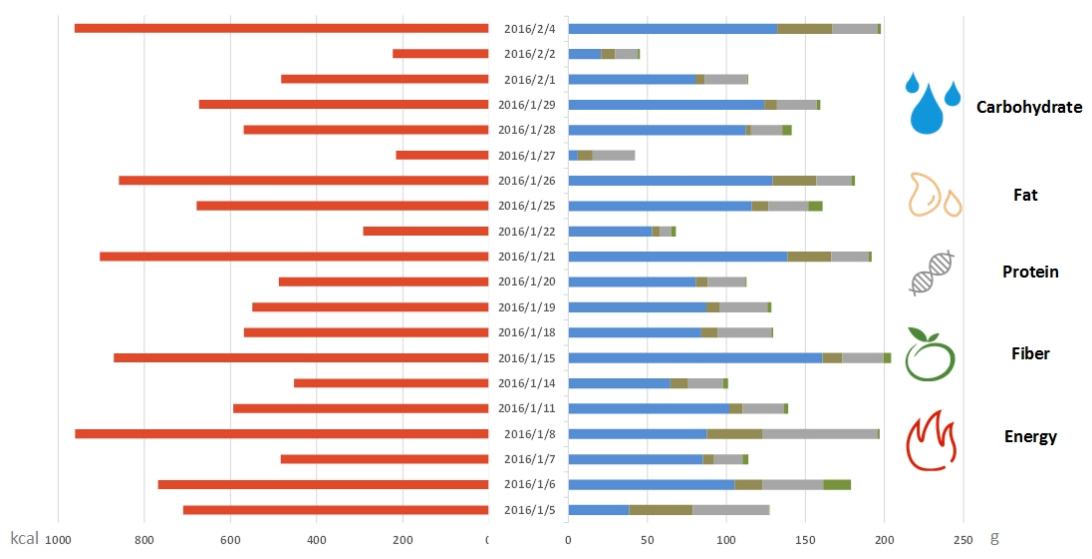
Example of system output: energy (left) and nutrient calculation results (right) for a sample individual. Blue: carbohydrate; orange: fat; gray: protein; green: fiber; red: energy (kcal).

Figure 7 shows the proxy indictors of dietary intake over the 20 working days from January 5 to February 4, 2016. The figure shows caloric intake at lunch (left) and nutrient intake (right) in the form of carbohydrate (blue), fat (orange), protein (gray), and fiber (green).

### Initial Indications of Nutrient Calculation

To examine our nutrient calculation performance, we selected 3 Chinese food dishes for comparison analysis. The 3 dishes were garlic puree cooked pork leg, dry-fried string beans, and roast lamb, which were measured by chemical methods in a previous study [22]. Given a dish of Chinese food (weight = 100 g), we calculated its nutrient content as C={*c*_1_, *c*_2_, ..., *c*_n_}, where *c*_x_ is a nutrient such as energy, protein, fat, or carbohydrate. We used mean absolute percentage deviation (MAPD) [23] to express the difference between our calculation results and the measurement M={*m*_1_, *m*_2_, ..., *m*_x_} obtained by chemical methods, where the Kjeldahl nitrogen method was applied to evaluate the protein content and the acid hydrolysis method was used to evaluate the fat content [22].

Table 1 shows the results of the comparison between our calculated nutrient values and the chemical measurements for the 3 selected Chinese cooked dishes. The MAPD of each nutrient value pair showed that our calculation method achieved comparable results to the chemical measurements, when considering the complexity and variety of Chinese food cooking.

**Table 1:** Nutrient calculation results compared with chemical measurements.

Chinese food dishes, ingredients and preparation method		Nutrient	Nutrient content measure		
			*c*_x_^a^	*m*_x_^b^	MAPD^c^ (%)
**Garlic puree cooked pork leg**					
	Pork, garlic, scallions, pepper; steaming	Energy (kcal)	288.1	253	13.87
		Protein (g)	25	22	13.64
		Fat (g)	20.9	18.3	14.21
		Carbohydrate (g)	0	0	—
**Dry-fried string beans**					
	String beans, minced pork, dried chilies, garlic, scallions; stir frying	Energy (kcal)	210.7	229	7.99
		Protein (g)	9.25	10.8	14.35
		Fat (g)	18.2	16.7	8.98
		Carbohydrate (g)	5.73	9	36.33
**Roast lamb**					
	Lamb, onion, cumin, oil; roasting	Energy (kcal)	133.4	162	17.65
		Protein (g)	19.4	21.5	9.77
		Fat (g)	6.2	8.1	23.46
		Carbohydrate (g)	0	0.8	100.00

^a^Nutrient content calculated in this study.

^b^Measurement obtained by chemical methods (Kjeldahl nitrogen method for protein content and acid hydrolysis method for fat content).

^c^MAPD: mean absolute percentage deviation.

## Discussion

### Principal Results

We developed a dietary management system to collect information on Chinese food using RFID technology. We applied the system to process real-world dietary data to test its feasibility. As shown in the preliminary results, the system can automatically record the Chinese foods carried by RFID-embedded plates and generate food frequency reports. With the support of the Chinese Recipe Database and China Food Composition Database, our dietary system broke down the cooked Chinese foods into their ingredients and nutrients. The system outputs included data on the frequency of the foods consumed and an overview of the composition of chosen foods, at both a population level and an individual level.

We compared the nutrients calculated for 3 selected Chinese foods with those measured by chemical methods. The result showed that the deviation was less than 20%. This deviation in calculated nutrients may have been caused by the instability in ingredient proportions and the change in food ingredient compositions according to location, season, and weather. The estimated weight of each dish and the ingredients may also result in deviation. However, comparison of 3 samples was insufficient to established a solid conclusion. More samples should be included for calculating *c*_x_ to compare against the reference standard set by chemical methods.

### Limitations

Our study had 3 main limitations. First, we estimated the weight of the food on each kind of plate by the type of plate and the food itself. We did not weigh exactly the consumed part on each plate. This may have affected the accuracy of our nutrient intake calculation. Second, the preparation methods used in Chinese cuisine affected the nutrient calculation results. Recent studies investigated the effects of cooking methods on specific foods [24,25]. We have not yet considered the effects of cooking method. Third, in Chinese food recipes, small amounts of auxiliary ingredients (eg, ginger and garlic) are commonly used. In this study, we ignored their nutrient contents in our calculation, which may have affected the nutrient analysis results. In our future work, we will consider the above factors such as precise weights and auxiliary ingredients to improve nutrient intake calculation accuracy. As the dietary management system was designed for clinical research and health consumers, user tests should also be conducted in future.

### Conclusions

In this study, we used an RFID-based system to track and record the foods and their corresponding weight eaten by our study group. We used 2 databases, a recipe database and a food composition database, to obtain the distribution of ingredients consumed by the group and to validate the calculated nutrients.

In future research, we will consider more factors (eg, preparation method, weight of the consumed portion, and auxiliary ingredients) in the nutrition calculation and improve the accuracy of our results. Additionally, we will combine the nutrition analysis with the consumer’s information, including health condition and demographic information such as age, sex, and birthplace. All of these efforts aim to provide more personalized dietary management.

## Abbreviations

ID: identification
MAPD: mean absolute percentage deviation
RFID: radio-frequency identification
